# Functional Characterization of the *Chlamydomonas reinhardtii ERG3* Ortholog, a Gene Involved in the Biosynthesis of Ergosterol

**DOI:** 10.1371/journal.pone.0008659

**Published:** 2010-01-11

**Authors:** Kristy M. Brumfield, James V. Moroney, Thomas S. Moore, Tiffany A. Simms, David Donze

**Affiliations:** 1 Department of Biological Sciences, Louisiana State University, Baton Rouge, Louisiana, United States of America; 2 Department of Biology, Xavier University of Louisiana, New Orleans, Louisiana, United States of America; Auburn University, United States of America

## Abstract

**Background:**

The predominant sterol in the membranes of the alga *Chlamydomonas reinhardtii* is ergosterol, which is commonly found in the membranes of fungi, but is rarely found in higher plants. Higher plants and fungi synthesize sterols by different pathways, with plants producing cycloartenol as a precursor to end-product sterols, while non-photosynthesizing organisms like yeast and humans produce lanosterol as a precursor. Analysis of the *C. reinhardtii* genome sequence reveals that this algae is also likely to synthesize sterols using a pathway resembling the higher plant pathway, indicating that its sterols are synthesized somewhat differently than in fungi. The work presented here seeks to establish experimental evidence to support the annotated molecular function of one of the sterol biosynthetic genes in the *Chlamydomonas* genome.

**Methodology/Principal Findings:**

A gene with homology to the yeast sterol C-5 desaturase, *ERG3*, is present in the *Chlamydomonas* genome. To test whether the *ERG3* ortholog of *C. reinhardtii* encodes a sterol C-5 desaturase, *Saccharomyces cerevisiae ERG3* knockout strains were created and complemented with a plasmid expressing the *Chlamydomonas ERG3*. Expression of *C. reinhardtii ERG3* cDNA in *erg3* null yeast was able to restore ergosterol biosynthesis and reverse phenotypes associated with lack of *ERG3* function.

**Conclusions/Significance:**

Complementation of the yeast *erg3* null phenotypes strongly suggests that the gene annotated as *ERG3* in *C. reinhardtii* functions as a sterol C-5 desaturase.

## Introduction

Sterols are isoprenoid-derived molecules found in the membranes of eukaryotic organisms and have been shown to play an important role in membrane fluidity and permeability [1]–[4]. While sterols are essential components of eukaryotic membranes, the specific sterols found in different organisms vary [5]. Cholesterol is the predominant sterol in vertebrates and ergosterol is the most common sterol found in fungi. Plants have a variety of sterols including sitosterol, 24-methyl cholesterol and stigmasterol [6].

**Figure 1:**
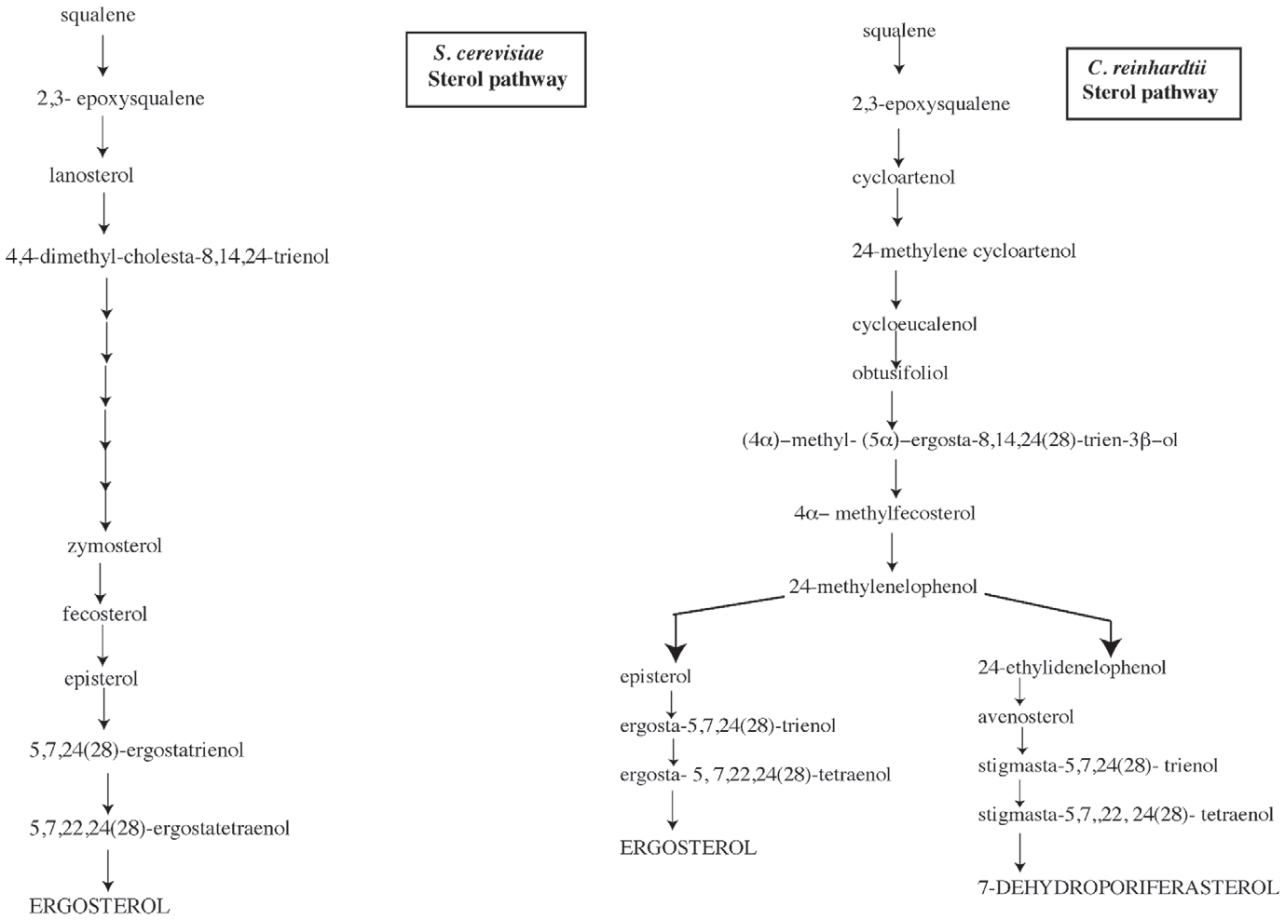
Schematic diagram of the putative pathway of ergosterol biosynthesis in *S. cerevisiae* and *C. reinhardtii*.

Two major pathways for sterol biosynthesis have been found in eukaryotes. Fungi and vertebrates synthesize sterols with lanosterol as an intermediate (Fig. 1), while plants synthesize sterols using cycloartenol as an intermediate [5]. In these pathways the biosynthetic steps from isopentanyl PP to squalene epoxide are the same. However, the cyclization of squalene epoxide is where the two pathways diverge producing either cycloartenol or lanosterol [7]. Nes *et al*., have identified cycloartenol in fungal-like organisms *Prototheca wickerhamii* and *Dictyostelium discoidium* that have been presumed to be descendents of algae after the evolutionary loss of the chloroplast [8]. It is now scientifically accepted that red algae, green algae and diatoms make cycloartenol while dinoflagellates have been reported to make lanosterol [9], [10].

In the green alga *C. reinhardtii*, the predominant sterols are ergosterol and 7-dehydroporiferasterol [11]. These two sterols are commonly found in fungi, but not so often with higher plants. However, bioinformatics evidence supports the idea that *C. reinhardtii* uses the cycloartenol pathway, as genes coding for orthologs of cycloartenol cyclase and cyclopropyl isomerase, two key enzymes in the cycloartenol pathway, are found in the *C. reinhardtii* genome [12] (Brumfield and Moroney, unpublished results). So while *C. reinhardtii* synthesizes ergosterol, a sterol normally associated with the fungal biosynthetic pathway, it appears to use a pathway that more closely resembles that of higher plants. Earlier studies of ergosterol deficient mutants in *C. reinhardtii* provide evidence for the final few steps of ergosterol biosynthesis in this alga [13], [14]. However, the steps from squalene epoxide to ergosta- 5,7,24 (28)-trienol have not been determined. A goal of this work is to further elucidate the sterol biosynthetic pathway in *C. reinhardtii*.

With the publication of the *Chlamydomonas* genome, genes likely to be associated with sterol biosynthesis in *C. reinhardtii* can be identified [12]. While *C. reinhardtii* offers many advantages as an experimental organism, it is still difficult to obtain targeted knock-out mutants of a desired gene. In addition, the fact that mutants earlier in the pathway have not been identified implies that a complete loss of sterol might be lethal to the alga under normal growth conditions. An alternative approach is to use *Sacchromyces cerevisiae* to study the function of genes involved in sterol biosynthesis in *C. reinhardtii*. The post-squalene biosynthesis pathway of ergosterol in yeast has been well defined [15], [16]. Enzymes of the ergosterol biosynthetic pathway have been found to be major targets for drug interactions, and a number of antifungal drugs on the market were derived specifically to target ergosterol biosynthesis [17]–[19]. Much of what is known about the sterol pathway in *Arabidopsis* has been worked out by complementing yeast strains defective in specific steps of sterol biosynthesis with the *Arabidopsis* ortholog [20]. We have previously used this approach to study phospholipid biosynthesis in *C. reinhardtii* [21], [22].

**Figure 2:**
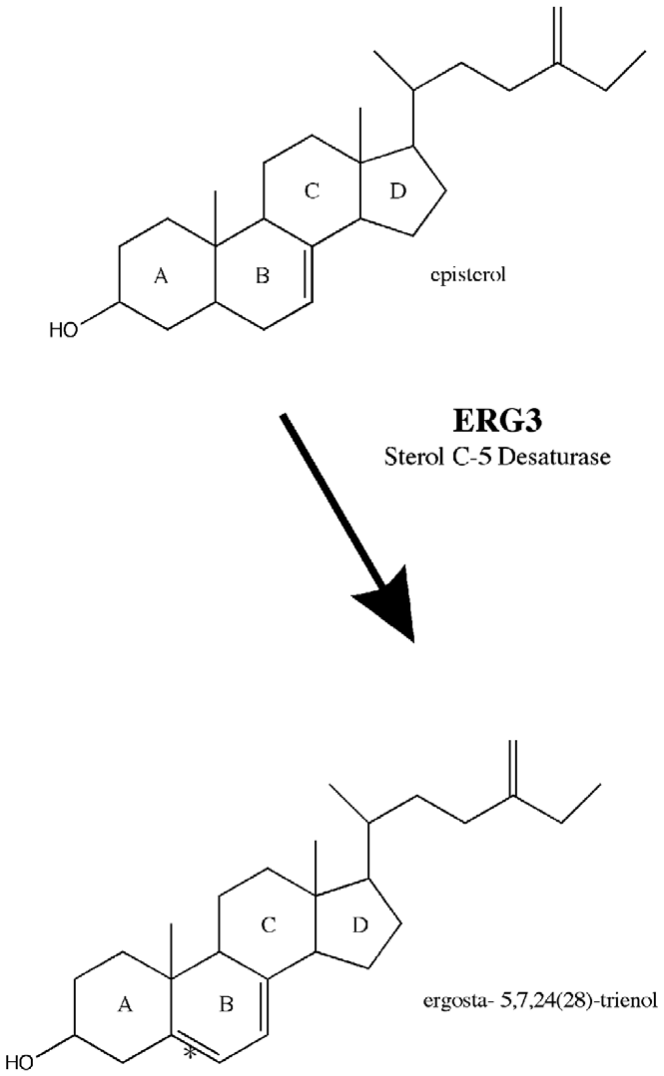
Schematic diagram of the reaction catalyzed by Erg3p in yeast. Erg3p is responsible for introducing a double bond at the C-5 carbon (denoted by the star) of the B-ring of episterol to produce ergosta- 5,7,24(28)- trienol. This step is the second to last step in the biosynthetic pathway to ergosterol.

One gene essential to ergosterol biosynthesis is *ERG3*, which encodes the C-5 sterol desaturase responsible for introducing a double bond at C-5 in the B ring of episterol [23] (Fig. 2). This enzyme is sensitive to cyanide and requires iron as well as molecular oxygen for its activity [15]. *ERG3* in yeast is also associated with NAD(P)H-cytochrome *b*/cytochrome *b*_5_ [23]. It has been previously demonstrated that *ERG3* is required for the breakdown of respiratory substrates when cells are heme deficient [24]. Deletion of *ERG3* in *S. cerevisiae* produces haploid cells that overaccumulate the ergosterol precursor, episterol [25]. Episterol and ergosta-7, 22-dien- 3-beta-ol are reported as possible substrates for the C-5 sterol desaturase [26]. Cloning and sequencing of *ERG3* has been reported in several model systems including *Arabidopsis thaliana* [27] *and Homo sapiens* [28]. In yeast, *ERG3* is a non-essential gene except under environments of heme-deficiency [24]. It has been suggested that Erg3 protein is a critical target in ergosterol biosynthesis [29], and when other ergosterol biosynthetic enzymes are mutated, *ERG3* expression is directly affected and regulated by these mutations [15].

In this study, we begin to characterize the function of *ERG3* in *C. reinhardtii* by complementation in *S. cerevisiae* ergosterol mutants. Questions remain as to whether algae produce sterols for other purposes, such as hormonal regulators [30], or if they function strictly for membrane integrity. Understanding the dynamics of this pathway may provide further evidence for hormonal regulation in *C. reinhardtii*, and the important cellular functions that may arise from the production of these sterols and their biosynthetic precursors.

## Results

### Sequence Analysis of the ERG3 Gene

A comparison of the *Saccharomyces cerevisiae ERG3* to the *Chlamydomonas* genome revealed several genes with significant protein sequence homology. The gene with the highest homology had a 21% sequence identity to the *ERG3* sterol desaturase of yeast. This *Chlamydomonas* gene also had a high similarity to *ERG3* genes annotated in higher plants. The gene with the next highest homology had a sequence identity of only 16%, and appears to be more closely related to the sequence of the yeast *ERG25* methyl sterol oxidase. Based on both the higher similarity of the 21% homologous gene to yeast and plant *ERG3*, and the higher similarity of the 16% homologous gene to *ERG25*, we chose the former gene for further analysis, and tentatively annotated it as *Chlamydomonas ERG3* (Accession No. XP_001701457).

**Figure 3:**
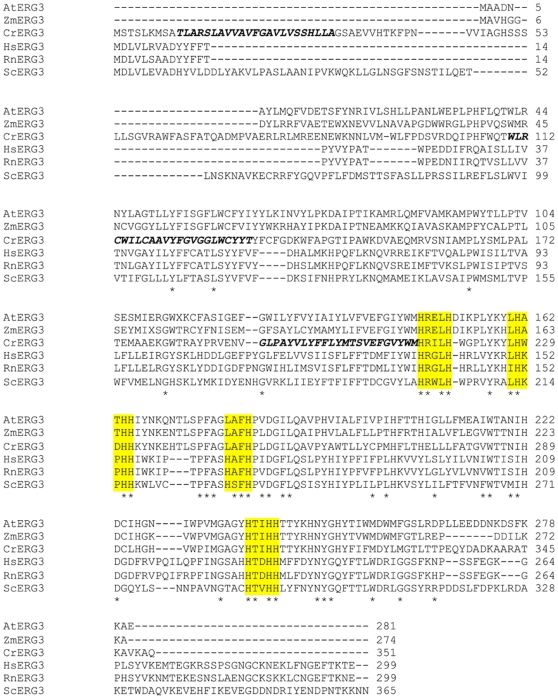
Amino acid sequence alignment of Sterol C-5 desaturase in different organisms. *Arabidopsis thaliana* (AtERG3) NCBI Accession Number CAA62079; *Zea mays* (ZmERG3) ACG38774; *Chlamydomonas reinhardtii* (CrERG3) XP_001701457; *Homo sapiens* (HsERG3) BAA33729; *Rattus norvegicus* (RnERG3) NP_446094; *Sacchromyces cerevisiae* (ScERG3) NP_013157. Conserved amino acid sequences shared by all organisms are denoted by a star. The highlighted area corresponds to the putative histidine-containing metal binding domain. Dashed lines indicate gaps in the alignment. The bold, italicized font corresponds to the three putative transmembrane spanning regions of the *C. reinhardtii ERG3* protein.

**Figure 4:**
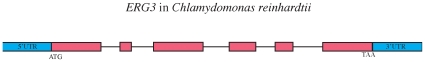
Schematic diagram of sterol C-5 desaturase in *C. reinhardtii* as annotated in the JGI *Chlamydomonas* Genome Version 4.0. ERG3 has six exons and five introns.

In yeast, *ERG3* is a C-5 sterol desaturase that adds a double bond in the ring structure of episterol to produce in the ergosterol biosynthetic pathway [31] (Fig. 2). In the proposed pathway for ergosterol biosynthesis in *C. reinhardtii* (Fig. 1), *ERG3* would catalyze the conversion of episterol to ergosta- 5,7,24 (28)-trienol. *ERG3* in *C. reinhardtii* is located on chromosome 16 of the genome [12], and the predicted amino acid sequence of *ERG3* aligns well with other C-5 sterol desaturases (Table 1 and Fig. 3). The *ERG3* gene in *C. reinhardtii* encodes a protein of 351 amino acids [12]. The cDNA is approximately 1.1kb long and is composed of six exons according to Version 4.0 of the Chlamydomonas Genome [12] (Fig 4). Like other known Erg3 proteins, the homologous gene in *C. reinhardtii* codes for four putative histidine metal binding domains [28] (Fig. 3). A genome database (http://genomeportal.jgi-psf.org/Chlre4/Chlre4.home.html) predicts that Erg3 protein has 3 transmembrane helices. The first helix is thought to be made up of amino acids 10–32, while the second and third helices correspond to amino acids 110–132 and 191–213 [12], [32], respectively (Fig. 3).

**Table 1:** Alignment scores among sterol C-5 desaturases of various organisms generated from ClustalW.

	AtERG3	ZmERG3	CrERG3	HsERG3	RnERG3	ScERG3
AtERG3	-	65	46	27	27	24
ZmERG3	65	-	48	24	25	24
CrERG3	46	48	-	19	19	21
HsERG3	27	24	19	-	82	43
RnERG3	27	25	19	82	-	44
ScERG3	24	24	21	43	44	-

*Arabidopsis thaliana* (AtERG3); *Zea mays* (ZmERG3); *Chlamydomonas reinhardtii* (CrERG3); *Homo sapiens* (HsERG3); *Rattus norvegicus* (RnERG3); *Saccharomyces cerevisiae* (ScERG3).

### Deletion of ERG3 by Homologous Recombination

**Figure 5:**
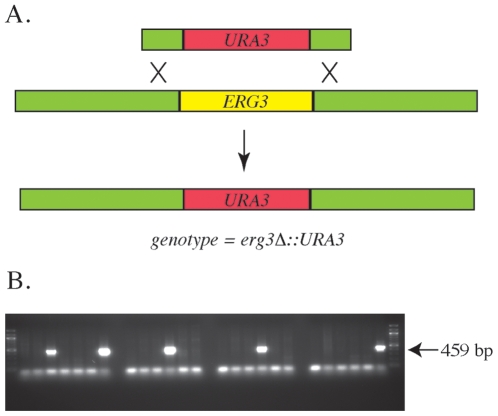
Deletion of the ERG3 gene in yeast. (A) Schematic diagram of homologous recombination strategy used to delete *ERG3* in *Sacchromyces cerevisiae*. PCR primers were designed to amplify the *URA3* marker gene containing the flanking regions of *ERG3*. (B) Ura^+^ isolates were screened by PCR to verify proper integration of *URA3* and deletion of *ERG3*. Only correctly integrated isolates would show a 459 base pair product using primers internal to *URA3* and upstream of the flanking homology. Stars denote the positive isolates selected for further analysis. PCR was also used to verify the downstream end of flanking region homology with *URA3* (data not shown).

While *ERG3* from *Chlamydomonas* aligned well with putative plant *ERG3* genes (Fig. 3), its alignment with the characterized *ERG3* protein of yeast showed a lesser degree of identity and similarity. To provide evidence that *Chlamydomonas ERG3* functions as a C-5 desaturase, we designed experiments to complement a yeast *erg3*Δ mutant. To generate the null mutation in yeast, the *ERG3* gene was knocked out by homologous recombination and replaced with the *URA3* marker in a diploid yeast strain (Fig. 5A). PCR analysis was used to identify positive yeast recombinants that possessed the *ERG3* mutation, and these isolates were sporulated to obtain haploid *erg3*Δ strains (Fig. 5B). Yeast lacking a functional *ERG3* are viable, as the gene is non-essential under aerobic conditions [33]. In addition, yeast lacking *ERG3* have an increased sensitivity to cycloheximide as previously described [34]. Haploid isolates obtained from yeast *ERG3* knockouts demonstrated increased sensitivity to cycloheximide when grown under optimal conditions in rich media.

### Complementation of ERG3 Mutants

**Figure 6:**
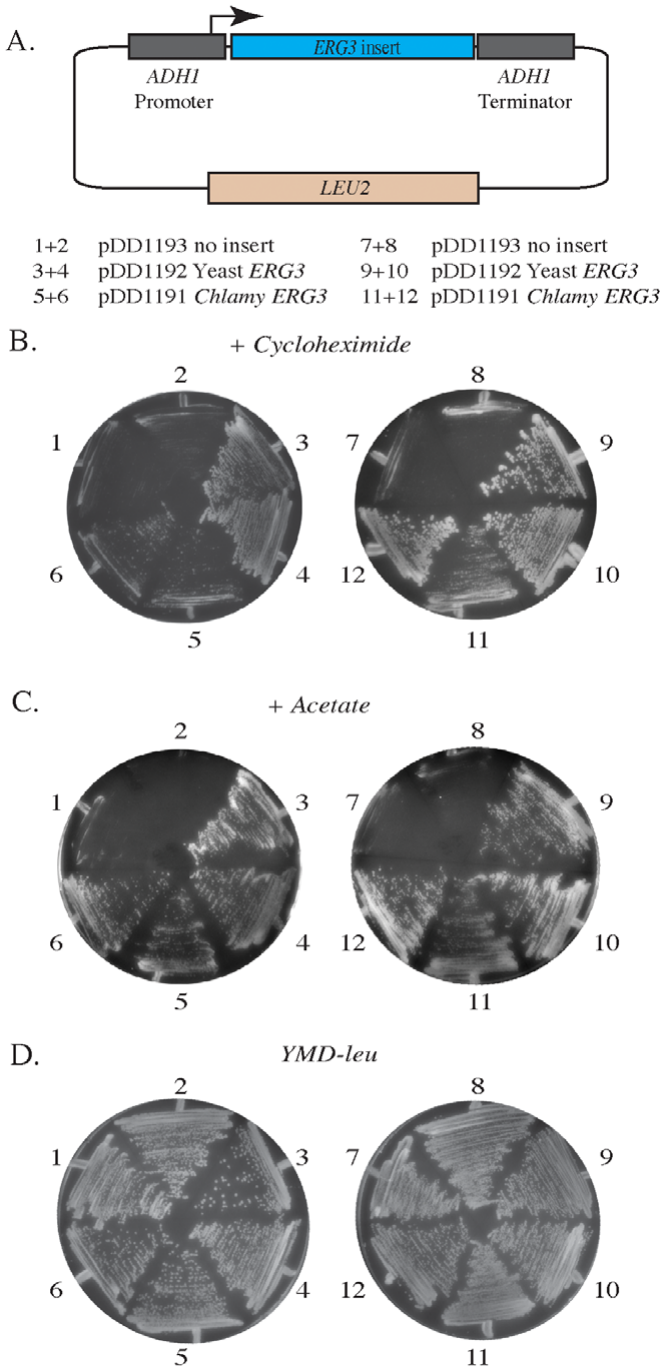
Phenotypic complementation of yeast *erg3*Δ strains. Known phenotypes of yeast *erg3* null mutants (hypersensitivity to cycloheximide and inability to grow on acetate) were complemented by plasmids expressing either *S. cerevisiae ERG3* or *C. reinhardtii ERG3* cDNA. A) *S. cerevisiae ADH1* promoter plasmid used to express *ERG3* genes. Haploid yeast carrying the *erg3Δ::URA3* allele were transformed with the designated plasmids and plated on dextrose minimal media lacking leucine (YMD minus Leu) to select for transformants. Single colony isolates were then re-streaked on minimal media lacking leucine and containing cycloheximide (0.13 µg/mL) (B) minimal media lacking leucine and containing acetate as sole carbon and energy source (C), and on YMD lacking as overall growth control (D). Yeast mutant for *ERG3* and containing the vector plasmid lacking an insert cannot grow on cycloheximide or acetate, while cells containing the plasmid expressing either yeast or *C. reinhardtii ERG3* are able to grow. Each pair of plates represents *erg3*Δ yeast sporulated from two independently isolated diploid knockout isolates, 1+2 are DDY4259 and DDY4260 transformed with the vector only, 3+4 are DDY4261 and DDY4262, the same strains transformed with *ADH1*-yeast *ERG3*, and 5+6 are DDY4263 and DDY4264 with *ADH1-C.reinhardtii ERG3*. For the second panel, 7+8 are DDY4253 and DDY4254 transformed with the vector only, 9+10 are DDY4255 and DDY4256 transformed with *ADH1*-yeast *ERG3*, and 11+12 are DDY4257 and DDY4258 with *ADH1-C.reinhardtii ERG3*.

To test the function of the *C. reinhardtii ERG3* gene, complementation experiments using yeast haploid strains deficient in *ERG3* were conducted. Yeast *erg3*Δ strains display a marked hypersensitivity to low non-lethal levels of cycloheximide [34]. In these experiments, plasmids expressing the yeast *ERG3* ORF, *C. reinhardtii ERG3* ORF and the empty vector were transformed into the *erg3*Δ knockout strains (Fig. 6A and Table 2). The selectable marker *LEU2* was used to select for transformants on minimal media lacking leucine (Fig. 6A), and Leu+ isolates were then screened for resistance to low levels of cycloheximide (Fig. 6B). Mutant *erg3*Δ strains transformed with the pDD1193, the empty vector, remained hypersensitive to cycloheximide, as they displayed very little growth on plates containing the drug (Fig. 6B). Transformants containing plasmid pDD1192 expressing the yeast *ERG3* ORF were completely resistant to low levels of cycloheximide (Fig. 6B). Transforming the mutants with pDD1191, expressing the *C. reinhardtii ERG3* ORF, showed enhanced resistance to the drug, demonstrating that the algal gene could complement the loss of the yeast *ERG3* (Fig. 6B).

**Table 2:** Yeast strains used in this work.

Strains	Genotype	Source
DDY 4259	*MAT ADE2 his3 leu2 LYS2 trp1 ura3 erg3Δ::URA3 pDD1193*	This study
DDY 4260	*MAT* **a** *ADE2 his3 leu2 lys2Δ trp1 ura3 erg3Δ::URA3 pDD1193*	This study
DDY 4253	*MAT ADE2 his3 leu2 LYS2 trp1 ura3 erg3Δ::URA3 pDD1193*	This study
DDY 4254	*MAT ADE2 his3 leu2 LYS2 trp1 ura3 erg3Δ::URA3 pDD1193*	This study
DDY 4261	*MAT ADE2 his3 leu2 LYS2 trp1 ura3 erg3Δ::URA3 pDD 1192*	This study
DDY 4262	*MAT* **a** *ADE2 his3 leu2 lys2Δ trp1 ura3 erg3Δ::URA3 pDD1192*	This study
DDY 4255	*MAT* **a** *ade2 his3 leu2 lys2Δ trp1 ura3 erg3Δ::URA3 pDD1192*	This study
DDY 4256	*MAT ADE2 his3 leu2 LYS2 trp1 ura3 erg3Δ::URA3 pDD1192*	This study
DDY 4263	*MAT ADE2 his3 leu2 LYS2 trp1 ura3 erg3Δ::URA3 pDD1191*	This study
DDY 4264	*MAT* **a** *ADE2 his3 leu2 lys2Δ trp1 ura3 erg3Δ::URA3 pDD1191*	This study
DDY 4257	*MAT* **a** *ade2 his3 leu2 lys2Δ trp1 ura3 erg3Δ::URA3 pDD1191*	This study
DDY 4258	*MAT ADE2 his3 leu2 LYS2 trp1 ura3 erg3Δ::URA3 pDD1191*	This study

Previous work had also reported that yeast *ERG3* mutants are viable but cannot grow on non-fermentable carbon sources [24], [33], [35]. We used this phenotype as a second test of the ability of the *Chlamydomonas ERG3* to complement yeast *erg3*Δ strains. Consistent with the earlier observations, transformants expressing *C. reinhardtii* or yeast *ERG3* ORFs are able to grow on acetate as a sole carbon source, while the mutants transformed with the empty vector control cannot grow (Fig. 6C). All strains (Table 3) showed normal growth on minimal dextrose media minus leucine (Fig. 6D).

**Table 3:** Plasmids used in this study.

Plasmid	Description	Source
pDD 1191	1.1 kb Hind III fragment containing *ERG3* open reading frame from *C. reinhardtii* inserted into pDD 1193	This study
pDD 1192	1.1 kb Hind III fragment containing *ERG3* open reading frame from *S. cerevisiae* inserted into pDD 1193	This study
pDD 1193	*LEU2* marked *S. cerevisiae ADH1* promoter vector.	This study

**Figure 7:**
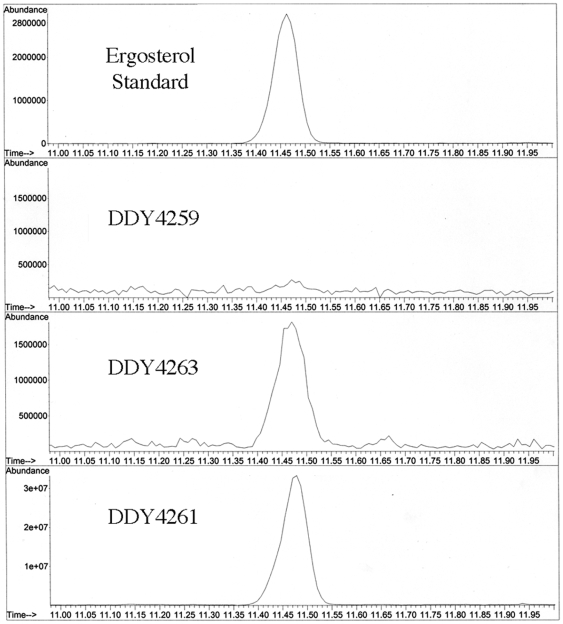
GC/MS data. Total ion chromatographs were analyzed to look specifically for the 100% ion of ergosterol. Mass ion 363 gives the best indication of the presence or absence of ergosterol in the samples. (A) Ergosterol standard, (B) Lipid extract from DDY4259 vector control, (C) DDY4263 expressing *C.reinhardtii ERG3*, (D) DDY4261 expressing *S. cerevisiae ERG3*.

To further characterize the degree of complementation, qualitative GC/MS analysis was performed to verify the presence of ergosterol in the complemented yeast transformants. Cultures of *erg3*Δ yeast expressing *S. cerevisiae ERG3* or *C. reinhardtii ERG3* were grown to similar cell densities, collected, and lipids were extracted and derivatized for analyses. Selected ion monitoring for ion 363 of ergosterol was used to search the total ion chromatograph for ergosterol in the samples (Fig. 7). When analyzing the total ion chromatograph data for specific ions of ergosterol, the knockout strain with the vector control was found to have a marked reduction in the level of ergosterol in the membranes compared to the strains complemented with the *ERG3* ORF from *C. reinhardtii* and *S. cerevisiae* (Fig. 7). Gas chromatography data contained peaks with retention times for the ergosterol standard, DDY 4263, and DDY 4261 respectively at 11.46, 11.46, and 11.47 minutes. The mass ions coinciding with these specific retention times gives spectral data with ions 468, 363, 337, and 253 *m/z* as previously described as ergosterol mass ions by Griffiths *et al* [36]. These experiments demonstrate that expression of *ERG3* from *C. reinhardtii* can restore ergosterol biosynthesis in *erg3*Δ yeast.

## Discussion

Ergosterol is the major sterol found in membranes of *C. reinhardtii*. However, the specific genes involved in sterol biosynthesis in *Chlamydomonas* have not yet been fully characterized. Several sterol mutant strains were previously identified in *C. reinhardtii* by selecting for resistance to the polyene antibiotic, nystatin [13], [37]. Polyene antibiotics function by forming complexes with the sterols in the membrane and decreasing membrane selective permeability, thereby causing cell death [38]. This method of selection was used to identify ergosterol mutants and allow researchers to delineate the final steps of ergosterol biosynthesis in *C. reinhardtii*, namely from ergosta- 7, 24(28)- dienol or 5- dehydroepisterol to ergosterol (Fig. 1). However, until recently very little was known about the steps from squalene epoxide to ergosta- 7, 24(28)-dienol. With the publication of the *Chlamydomonas* genome [12], we are now able to identify genes that may play a role in sterol biosynthesis in *C. reinhardtii*. Analysis of the genome indicates that the *C. reinhardtii* sterol biosynthetic pathway from squalene epoxide to episterol most closely follows the higher plant pathway. This means that *C. reinhardtii* is synthesizing ergosterol differently than yeast although many of the individual steps and enzymes are homologous. Yeast genetics allows not only the study of the function of yeast genes, but is also useful for cross-species complementation studies, and unlike *C. reinhardtii*, several ergosterol mutants have been identified in yeast, and their phenotypes have been well documented [16], [39]. We chose *ERG3* for further study, as specific phenotypes previously characterized in yeast *erg3* mutants are readily complemented.

*ERG3* encodes a sterol C-5 desaturase, and belongs to the fatty acid hydroxylase superfamily. This superfamily of proteins includes C-5 sterol desaturases as well as C-4 sterol methyl oxidases [32], [40], [41]. This family of proteins possesses four putative iron-binding domains [28]. In yeast, loss of *ERG3* function leads to an apparent loss of ergosterol in the membrane and an increase in the closely related precursor, episterol. The replacement of ergosterol with episterol in yeast leads to increased sensitivity to cycloheximide and an inability to grow with acetate as a sole carbon source [24]. The efficient gene replacement techniques used in *Sacchromyces cerevisiae,* has allowed for the creation of mutant strains in which the *ERG3* gene has been replaced with *URA3* [42], [43], providing an experimental tool for complementation studies.

Complementation of yeast knockout strains has been used successfully to identify genes involved in sterol biosynthesis in *Arabidopsis* [20]. The yeast *Erg25* (sterol 4α-methyloxidase) [44], *Erg24* (sterol C-14 reductase) [45], *Erg2* (Δ8-Δ7 sterol isomerase) [46] and *Erg3* [47] mutants have all been complemented by the corresponding *Arabidopsis* genes, even though the homology between the yeast and *Arabidopsis* proteins was fairly low and the natural substrate in *Arabidopsis* differs somewhat from that of the yeast pathway. These previous results with *Arabidopsis* suggested that *Chlamydomonas* genes encoding homologous proteins might also be identified by complementation in yeast. In the work described here we were able to confirm the annotation of the *C. reinhardtii ERG3* ortholog as a C-5 sterol desaturase by both phenotypic complementation and direct biochemical analysis.

While the *C. reinhardtii ERG3* ORF does function in yeast, it must be noted that it does not complement as fully as the yeast *ERG3* gene. There are several explanations for this. Traditionally, 2 micron plasmids have been used in yeast for heterologous complementation, in order to increase the copy number of the plasmid and possibly circumvent issues regarding variations in protein stability, codon usage, and enzyme activity across species [48]. This study uses a moderate copy number plasmid. Secondly, yeast and *C. reinhardtii* undergo two very different biochemical pathways for the production of sterols. Yeast use the traditional MVA (IPP) pathway for the synthesis of sterols, while *C. reinhardtii* utilizes the DOXP pathway for the synthesis of sterols [49]. Evolutionary differences across species allow for several variations in the ergosterol biosynthetic pathway. For example, *C. reinhardtii* and *S. cerevisiae* produce precursors to ergosterol by two independent pathways so therefore similar enzymes can often catalyze somewhat different reactions [27], [49]. Finally, ergosterol biosynthesis is a very metabolically taxing process that requires molecular oxygen, sources of energy, and optimal temperatures [50]. Despite these potential complications, complementing the yeast *ERG3* null mutation with the *ERG3* gene from *C. reinhardtii* results in the production of ergosterol, and increased survival of the cells during exposure to cycloheximide and growth on acetate.

Sterol biosynthesis is very intricate in nature, and while several *ERG3* orthologs from different eukaryotic species have been identified and characterized, *ERG3* mutants in *Chlamydomonas* have not been created. Previous studies using random mutagenesis in *Chlamydomonas* identified mutants in the last two steps of ergosterol biosynthesis but not in the earlier steps of sterol biosynthesis, which includes *ERG3*. Presently, there are no consistently reliable methods to knock-out specific genes in *Chlamydomonas* although new methods are being developed. While bioinformatics as well as functional complementation gives insight into the function of a gene and its corresponding proteins, knockout experiments in the future will allow for a more complete characterization of the gene, as a targeted mutation of the gene we have annotated as *ERG3* would allow confirmation of the C-5 sterol desaturase activity observed by complementation in yeast.

Elucidation of the ergosterol biosynthetic pathway in *C. reinhardtii* is of considerable interest and importance, as plant and algal sterols are used as dietary supplements, and as components of cosmetics and pharmaceuticals [51]. For example, experimental evidence has shown that uptake of plant sterols can help to reduce cholesterol levels in humans [52], [53]. The similarity of the *C. reinhardtii ERG3* gene with that of higher plants indicates that the *Chlamydomonas* system may provide an excellent model system in which to study plant sterol biosynthesis. Better understanding of this pathway will lead to the development of genetically modified strains in *Chlamydomonas* that may allow for overproduction of specific ergosterol precursors.

## Materials and Methods

### Yeast Competent Cell Preparation and Transformation

For complementation experiments, yeast were transformed with plasmids expressing *ERG3* from *Chlamydomonas* and *ERG3* from yeast using the protocol of Geitz et al [54]. For the construction of *erg3*Δ strains, single yeast colonies were inoculated and grown overnight. In the morning, cultures were diluted to an optical density of 0.2 (A_600_), and cells were grown until reaching an approximate optical density of 0.7. Cells were harvested by centrifugation (2000×g for 5 minutes) at room temperature, and the cell pellet was resuspended in 1 mL of 1xTEL (10 mM Tris-HCl pH 7.5, 1 mM EDTA pH 8.0, 100 mM LiAc) per 10 mL culture volume and rocked overnight at room temperature.

### Yeast Strains and Growth

Yeast strains were constructed by homologous recombination using primers 5′- TGCATTTGTAAAAAAAGATAAAAGAAAAATATTCGTCTAGATTTGAGA TGCAG ATTGTACTGAGAGTGC- 3′ (forward) and 5′- TCTTGAACGTGAAAGAAAGAA AAAAGATGAGACAAACAAGGCAACCGTA TCTCCTTACGCATCTGTGCGG-3′ (reverse) to amplify the yeast *URA3* gene flanked by *ERG3* sequence. This PCR product was transformed into yeast as described above, Ura+ colonies were isolated, and *erg3*Δ isolates verified by PCR analysis. Deletion of *ERG3* was done in DDY2, a diploid version of *S. cerevisiae* W303), which was then sporulated to haploid (see below). Yeast were cultured in YPD (1% yeast extract, 2% peptone, 2% dextrose) or YMD (synthetic minimal medium, 2% dextrose, U. S. Biologicals catalog # Y2025) supplemented with required amino acids and nucleotides [55]. Unless otherwise stated, all yeast operations were carried out according to standard procedures [56]. To construct an *ADH1* promoter vector, the plasmid pOAD (gift from Dr. Stan Fields, University of Washington) was modified as follows. The *GAL4* coding sequence was removed by digestion with *Hind* III, and re-ligated to produce the *ADH1* promoter control vector pDD1193, and *ERG3* genes were coned into the resulting unique *Hind* III site. Expression of *S. cerevisiae* and *C. reinhardtii ERG3* cDNA from the *ADH1* promoter of yeast expression vector pDD1193 was carried out by culture of the transformed yeast strain in minimal media lacking leucine.

### Yeast Genomic DNA Extraction

Yeast genomic DNA was extracted by the Winston protocol [57].

### Tetrad Dissection

Heterozygous diploid *erg3*Δ::*URA3* cells were cultured on YPD plates, then transferred to spore plates. A colony-sized mass of cells was re-suspended in 6 µL Zymolase 100T (1 mg/mL, Sigma) for each sporulation and incubated for 2 minutes at room temperature. After incubation, 300 µL of water was added to each sample. Each sample solution was then spread along the upper portion of a petri dish and tetrads were separated with an Olympus B201 Dissecting scope. Each plate was incubated at 30°C, and cells were re-patched to a master plate. Each master plate was then replica plated to YMD dropout plates and mating tester lawns to determine genotype.

### cDNA Cloning

*Chlamydomonas Core Library*. The Chlamydomonas cDNA core library was purchased from the Chlamydomonas Center (http://www.chlamy.org/). The library was amplified using the host strain XL1 Blue MRF' (Stratagene catalog number 200301) according to the manufacturer's instructions at http://www.stratagene.com/lit/manuals/aspx (catalog number 236201).

The *S. cerevisiae ERG3* protein coding sequence (accession number NP_013157) was used to identify the *C. reinhardtii* EST with the best homology using the BLAST server (http://genomeportal.jgi-psf.org/Chlre4/Chlre4.home.html). Primers were designed based on the EST sequence in order to clone the coding region of cDNA from the core library described above. The primers used to clone the cDNA by PCR included the forward primer: 5′-GCGGCCGCCGATCGAAGCTTAATGTCAACCTCGCTCAAAATGA-3′ and the reverse primer: 5′-GCGGCCGCCGATCGAAGCTTTACTGCGCCTTGACGGCCT-3′.

### Sequence Analysis

Multiple sequence alignment programs from the European Bioinfomatics Institute EMBL-EBI server (http://www.ebi.ac.uk/clustalw/) were used for sequence analysis and alignment data. The Prosite database from EBI server (http://ca.expasy.org/prosite/) was used to identify putative consensus motifs for specific domains in *ERG3*. The *C. reinhardtii* genomic database provided information about the genomic sequence, intron-exon structure of *ERG3*, as well as information regarding potential membrane spanning regions of the protein coding sequence (http://genomeportal.jgi-psf.org/Chlre4/Chlre4.home.html).

### Drug Resistance Screen

Cycloheximide was purchased from Sigma Aldrich (catalog# C- 7698-1G). *S. cerevisiae* minimal media was prepared as previously described with the addition of cycloheximide [34] at a final concentration of 0.13 µg/mL from a stock solution of 2 mg/ml in 100% ethanol.

### Lipid Extraction

Yeast cells were grown to an optical density (A_600_) of 0.7-0.8. The cells were pelleted at 2000 rpm for 5 minutes and resuspended in 4 mL hot isopropanol (70°C) for 30 minutes. The cell wall was disrupted by vortexing with 0.5 mm glass beads for 2 minutes. Cell pellets were then resuspended in 4 mL hot isopropanol and lipids were extracted at 70°C for 2 hours. Next, 4 mL of chloroform/methanol (1∶2) and 2 mL of 1 M KCL were added to the lipid extracts, which were vortexed and centrifuged at 5000 rpm on a benchtop centrifuge for 5 minutes. The top aqueous phase was discarded and the previous extraction step was repeated three times. Then, 2 mL of water were then added to the samples. The samples were vortexed and centrifuged at 5000 rpm for 5 minutes, and the aqueous phase was discarded again. Lipids were dried under nitrogen and stored at −80°C until analysis. The lipid extraction protocol was derived from the Bligh and Dyer method [58].

### GC/MS Analysis

Ergosterol standard was purchased from Fluka. N,O-bis(trimethylsilyl)trifluoroacetamide (BSTFA) was used as the silylating reagent and was purchased from Sigma Aldrich (catalog #33084). 100 µL of BSTFA was added to dried lipid samples and heated to 60°C for 30 minutes. Derivatized samples were resuspended in 25 µL of dichloromethane and prepared for analysis. GC/MS was carried out on an Agilent 6890 gas chromatograph with an autosampler and Agilent 5973 mass selective detector. Then, 1 µL of sample was injected into a splitless system with a flow rate of 1 mL/min for the carrier gas, helium. The front inlet temperature was maintained at 250°C, while the mass selective detector line heater was at 280°C. The oven temperature was programmed to a final temperature of 300°C with an initial temperature of 100°C. The GC column, DB-5 ms, was 30 meters by 250 µm of internal diameter with a film thickness of 0.25 µm. Data was acquired by selective ion monitoring to detect ions at *m/z* 143, 211,337, 363, 468.
