# Correction: Functional Characterization of the *Chlamydomonas reinhardtii ERG3* Ortholog, a Gene Involved in the Biosynthesis of Ergosterol

**DOI:** 10.1371/journal.pone.0129189

**Published:** 2015-05-28

**Authors:** Kristy M. Brumfield, James V. Moroney, Thomas S. Moore, Tiffany A. Simms, David Donze

There is an error in Fig 2 of the published article. Please see the correct Fig 2 here.

**Fig 2:**
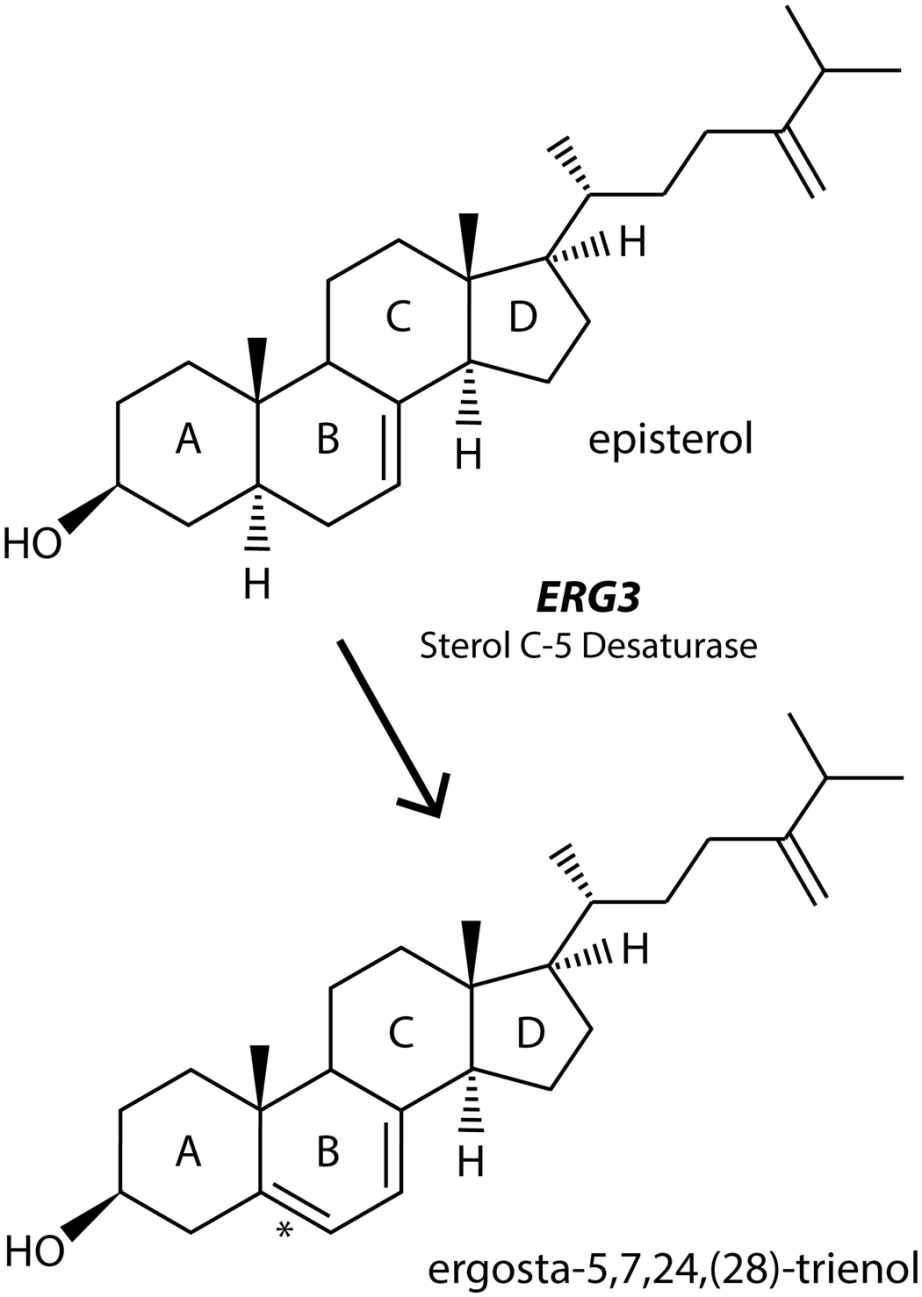
Schematic diagram of the reaction catalyzed by Erg3p in yeast. Erg3p is responsible for introducing a double bond at the C-5 carbon (denoted by the star) of the B-ring of episterol to produce ergosta- 5,7,24(28)- trienol. This step is the second to last step in the biosynthetic pathway to ergosterol.
